# RegulaTome: a corpus of typed, directed, and signed relations between biomedical entities in the scientific literature

**DOI:** 10.1093/database/baae095

**Published:** 2024-09-12

**Authors:** Katerina Nastou, Farrokh Mehryary, Tomoko Ohta, Jouni Luoma, Sampo Pyysalo, Lars Juhl Jensen

**Affiliations:** Novo Nordisk Foundation Center for Protein Research, University of Copenhagen, Blegdamsvej 3, Copenhagen 2200, Denmark; TurkuNLP Group, Department of Computing, University of Turku, Vesilinnantie 5, Turku 20014, Finland; Textimi, 1-37-13 Kitazawa, Tokyo, Setagaya-ku 155-0031, Japan; TurkuNLP Group, Department of Computing, University of Turku, Vesilinnantie 5, Turku 20014, Finland; TurkuNLP Group, Department of Computing, University of Turku, Vesilinnantie 5, Turku 20014, Finland; Novo Nordisk Foundation Center for Protein Research, University of Copenhagen, Blegdamsvej 3, Copenhagen 2200, Denmark

## Abstract

In the field of biomedical text mining, the ability to extract relations from the literature is crucial for advancing both theoretical research and practical applications. There is a notable shortage of corpora designed to enhance the extraction of multiple types of relations, particularly focusing on proteins and protein-containing entities such as complexes and families, as well as chemicals. In this work, we present RegulaTome, a corpus that overcomes the limitations of several existing biomedical relation extraction (RE) corpora, many of which concentrate on single-type relations at the sentence level. RegulaTome stands out by offering 16 961 relations annotated in >2500 documents, making it the most extensive dataset of its kind to date. This corpus is specifically designed to cover a broader spectrum of >40 relation types beyond those traditionally explored, setting a new benchmark in the complexity and depth of biomedical RE tasks. Our corpus both broadens the scope of detected relations and allows for achieving noteworthy accuracy in RE. A transformer-based model trained on this corpus has demonstrated a promising *F*1-score (66.6%) for a task of this complexity, underscoring the effectiveness of our approach in accurately identifying and categorizing a wide array of biological relations. This achievement highlights RegulaTome’s potential to significantly contribute to the development of more sophisticated, efficient, and accurate RE systems to tackle biomedical tasks. Finally, a run of the trained RE system on all PubMed abstracts and PMC Open Access full-text documents resulted in >18 million relations, extracted from the entire biomedical literature.

## Introduction

In the rapidly evolving field of Biomedical Natural Language Processing (BioNLP) and text mining, the development of novel, highly accurate deep learning–based methodologies [1] allows researchers to discover relations between biomedical entities. Relation extraction (RE) is a critical task that enables the identification of relations among named entities (NEs) such as genes, chemicals, and diseases. This process is essential for transforming unstructured text into structured data that can be used in both biological [2] and medical [3] applications.

The effectiveness of modern RE methodologies, particularly those leveraging the capabilities of pretrained transformer models tailored for the biomedical domain [4, 5], hinges on the size, quality, and scope of manually annotated corpora used for model fine-tuning. These corpora serve as training and evaluation resources, guiding the development of methods capable of accurate information extraction. However, a majority of currently available corpora for RE are constrained by focusing on relations at the sentence level [6–9] and/or relations between two types of entities only (e.g. gene–disease) [6, 7, 10, 11]. Such constraints limit the number of relations that can be effectively extracted from the literature.

Recognizing these limitations, the BioNLP community has begun to shift its focus toward the development of more comprehensive corpora that extend beyond the sentence level to encompass document-level annotations [11–14]. Standing out among them, the recent BioRED corpus [13], also tackles the issue of constrained scope, by having a broader coverage of eight different relation types among disease, gene, variant, and chemical entities. While there are event annotation corpora that primarily concentrate on proteins and related entities and offer many document-level event annotations [15–17], a noticeable absence remains in an RE corpus with the same properties.

In this work, we introduce RegulaTome, a corpus comprising 2521 documents with 16 961 document-level annotations, encompassing >40 types of relations—aligning with Gene Ontology (GO) [18, 19]—between 54 951 entities belonging to four different entity types. This corpus is specifically designed to illuminate the complex web of interactions between proteins and protein-containing entities, providing an invaluable resource for advancing the state of RE in the biomedical field. Using this corpus, we have trained a transformer-based model with commendable results on RE (*F*1-score = 66.6%) for such a difficult task. To achieve this, we have developed an RE system capable of multi-label extraction of these directed, typed, and signed relations from the entire biomedical literature. This work fills a critical gap in biomedical RE, offering a corpus and a system that allows the investigation of the complex interplay between proteins, protein-containing entities, and chemicals, which is foundational to understanding biological processes and disease mechanisms.

## Materials and methods

### The RegulaTome corpus

#### Targeted relation types

**Figure 1.**
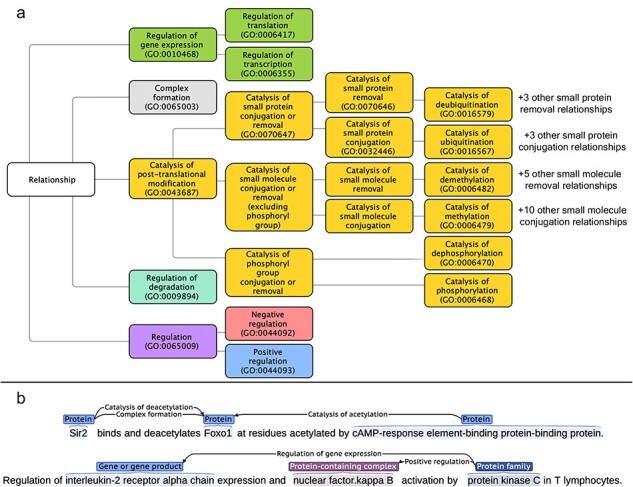
(a) Targeted relation types in RegulaTome and their relationship to each other: there are 43 relation types annotated in RegulaTome, mapped to the biological process sub-ontology of GO, and since GO lacks terms to collectively catalog all catalysis of small molecule conjugation or removal processes, we have decided to group catalysis of phosphoryl group conjugation or removal relations (i.e. catalysis of phosphorylation and catalysis of dephosphorylation) separately from the other catalysis of small molecule conjugation/removal relations, both because of their biological significance, and—most importantly—because we observed that these are discussed differently in the biomedical literature, (b) Illustration of relation representations in RegulaTome: multiple relations between a Protein (“Sir2”) and another Protein (“Foxo1”) participant are shown in the first sentence – an undirected Complex formation relation and a directed catalysis of deacetylation relation denoted with a left-to-right arrow, originating from “Sir2” with “Foxo1” as the target and a directed relation that has “Foxo1” as the target, this time originating from another Protein participant (“cAMP-response element-binding protein-binding protein”) and in the opposite direction denoted by a right-to-left arrow; relationships can arise between all entity types annotated, e.g. in the second sentence two directed relations (regulation of gene expression and positive regulation) originate from a Family (“protein kinase C”) participant and target a Protein participant and a Complex (“nuclear factor.kappa B”).

As mentioned earlier, the aim of this work was to allow the extraction of directed, typed, and signed relations for proteins, chemicals, protein-containing complexes, and protein families from the literature. As many relation types between biomedical entities can fulfill these criteria, in this section we provide a list of the relation types that we have decided to annotate. We have mapped and structured the relation type space on the “Biological Process” sub-ontology of GO [18, 19], a community-standard framework. The full list of targeted relation types, the GO term corresponding to each of them, and their direct parent in our sub-ontology of relations are given in Supplementary Section 1. Figure 1a shows an overview of the relationship tree, while Fig. 1b shows the relation representations within RegulaTome (for more details on the latter, please refer to the section “Named entity and relation annotation”).

#### Document selection for corpus annotation

The document selection process for the RegulaTome corpus consists of four steps:

1. ComplexTome corpus [20]: this corpus consists mostly of documents focusing on physical protein interactions. This corpus includes 137 abstracts with complex formation events from BioNLP ST 2009 datasets [15] and 450 abstracts and 400 paragraphs from full-text articles used as evidence to support interactions in the BioGRID [21], IntAct [22], and MINT [23] interaction databases. Moreover, this corpus contains 300 abstracts used for pathway annotation in the Reactome pathway knowledgebase [24], where regulatory relations are expected to be found in high prevalence. More details on the document selection of this corpus can be found in Mehryary *et al*. [20]. We reannotated all documents of ComplexTome to include all 43 relation types mentioned in Supplementary Section 1.
2. Posttranslational modification event extraction corpus: out of the 388 abstracts in this corpus originally annotated for the BioNLP 2010 workshop [16], we selected 234 based on each document containing at least one post-translational modification (PTM) event. We ignored the existing event annotations and completely reannotated the documents with relevant relations.
3. PTM triage set: a pool of 5548 publications from the Reactome database was generated by selecting those used to annotate pathways with at least one modification enzyme participant by the database curators [24]. We then went through the abstracts of these publications to select 500 of them if there was at least one catalysis of PTM relation of interest therein. The selection was done incrementally, with sets of 100 documents at a time, focusing on PTM relations with a lower number of total annotations every round to increase support for more relation types. Supplementary Section 2 provides more details on the selection process.
4. Reactome full-text excerpts set: a set of paragraphs from full-text articles used as evidence for pathway annotation in Reactome were selected if (i) they contained between 50 and 500 words—thus excluding documents with only titles or excessively lengthy paragraphs—(ii) the number of uniquely tagged NEs within these paragraphs—disregarding case sensitivity—exceeded three entities, and (iii) at least 30% of the entity mentions in each selected paragraph were forms not previously encountered in the documents of the corpus—to increase diversity. If a paragraph was chosen from a document for which its abstract is already included in our dataset, both the paragraph and the abstract were later assigned to the same subset (training, development, or test). There were 61 973 paragraphs from 21 941 papers fulfilling the criteria mentioned earlier, out of which we selected 500 for annotation. Similarly to the “PTM triage set”, selection was done in batches of 100 documents at a time. After initial observations, we tried to focus our annotations on specific sections of scientific papers, where most relations were expected to be mentioned. Supplementary Section 3 provides more details on the process.

#### NE and relation annotation

There are four NE types in this corpus: gene or gene products (Protein hereafter), chemicals (Chemical hereafter), protein-containing complexes (Complex hereafter), and protein families (Family hereafter). To annotate Complex entities, we have used the definition of the homonymous term from GO (GO:0032991). As for Family entities, we have only annotated entities that are evolutionarily related, using InterPro [25] as the main reference resource. Equivalent names of the same entities are systematically annotated to ensure evaluation accuracy [15].

In RegulaTome, we identified explicit mentions of >40 different relation types (Supplementary Section 1) and annotated those as either undirected (Complex formation) or directed (all other types) relations. Each candidate entity pair could receive multiple labels without any restrictions, and directed relations between the same entities could be bi-directional. Two examples of relation representations are shown in Fig. 1b.

Two experts in the field carried out the relation annotations for RegulaTome. An Inter-Annotator Agreement (IAA) analysis was performed to set uniform annotation standards and preserve the quality of annotations. This involved independently annotating a collection of the same abstracts in seven rounds. Four rounds of independent annotations were conducted on 80 documents from “ComplexTome” to establish the original annotation guidelines that acted as a guide to ensure annotators had a common understanding of them, aiding in the upkeep of high-quality annotations. Three additional rounds of IAA were conducted on 90 documents from the “Post-translational modification event extraction corpus.” This resulted in a set of updated guidelines for the entire corpus and a reannotation of all documents based on the updated set of rules. After each round, we measured the *F*1-score for IAA to evaluate the consistency of the annotations and the quality of the corpus. For detailed information on the annotation guidelines used to annotate NEs and relations in RegulaTome, we direct readers to the annotation documentation (available via Zenodo). The BRAT Rapid Annotation Tool [26] was used for the annotation of all documents in RegulaTome.

### RE system

We have extended the transformer-based RE system previously developed for binary RE [20] and created our current system that is capable of extracting the relation types presented in Supplementary Section 1 between all NE types mentioned earlier. The task of RE is cast as a multi-label classification problem, where the goal is to predict if a pair of candidate NEs in the input text has one, several, or no stated relations. For the undirected Complex formation relation, there is only one dimension in the decision layer of the neural network, whereas for each directed relation, there are two dimensions from the first occurring entity to the second occurring entity (i.e. left-to-right) and from the second occurring entity to the first occurring entity (i.e. right-to-left).

Similarly to the binary classification system upon which we built, our current system utilizes an architecture featuring a pretrained transformer encoder and a decision layer with a sigmoid activation function. The system can utilize pretrained language models available in the Hugging Face repository, accepts training, validation, and prediction data in BRAT standoff and a custom JSON format, and supports extensive hyper-parameters [including maximum sequence length (MSL), learning rate, number of training epochs, and batch size]. Evaluation metrics are calculated after each training epoch for hyper-parameter tuning. The system does not use any early stopping rule but is trained for a specified number of epochs and chooses the model weights that have yielded the highest *F*1-score.

The documents in our corpus, as is typical for biomedical documents, contain multiple candidate NE pairs and can be lengthy, often exceeding the maximum token capacity of transformer models. To clarify for the classifier which two candidate NEs are being considered for label prediction at a time, we encode these entities within the document using a masking approach, employing the model’s “unused” tokens for this purpose. We then tokenize the text and consider a window (a text snippet) around and including the two NEs (based on the MSL) and insert a [CLS] token at the beginning to signify the start of the snippet and a [SEP] token at the end of the input. For each candidate NE pair, we verify that the masked and tokenized snippet representing the pair does not exceed the specified MSL. If it meets this criterion, we proceed to create a machine-learning example for that pair. This example could be assigned one, multiple, or no labels for training or remain unlabeled for prediction. Since we do not employ any sentence boundary detection, we can train on and predict cross-sentence relations at the document level. Moreover, relying on a window that can always be fed to the transformer encoder allows us to effortlessly deal with long texts. If a candidate NE pair (i.e. a machine learning example) does not fit into the specified window size, it will be excluded in training and prediction and penalized in the evaluation of development and test sets (if the two NEs have any relations between them).

For more details on the implementation and the strategy for preprocessing, input representation, and example generation, refer to Mehryary *et al*. [20].

#### Experimental setup

We performed a document-based split of RegulaTome into separate training, development, and test sets for our experiments. We use grid search to find the optimal values of hyper-parameters. To minimize the impact of initial random weights on evaluation metrics in neural network models [27], we repeat each “experiment” four times and compare different experiments based on the average and standard deviation of the *F*1-scores. Each experiment consists of training an RE system (i.e. a neural network model) with the exact set of hyper-parameters but different initial random weights on the training set and evaluating the model on the development set. The hyper-parameter set that yields the highest average *F*1-score is chosen as the optimal, and the model with the highest *F*1-score in that experiment is selected for predicting the held-out test set and for large-scale execution of the RE system on biomedical literature. Therefore, the test set is used only once for evaluating our best model.

## Results and discussion

### Corpus statistics

RegulaTome is a corpus of high quality that contains 2521 documents with one paragraph each (1621 abstracts and 900 paragraphs from full-text articles) consisting of 611 999 words. This number of words in RegulaTome is comparable to Named Entity Recognition corpora, such as BC2GM [28] (569 912 words), and is much larger than other RE and event extraction corpora, such as BC5CDR [11] (360 373 words), BioRED [13] (143 246 words), and the BioNLP Shared Task 2011 REL (267 229 words) and EPI (253 628 words) corpora [17]. The corpus quality was assessed through seven rounds of IAA, which resulted in a final *F*1-score of 91% for IAA of all relation types. RegulaTome includes a total of 16 961 relations, with 6463 of them being Complex formation (∼38%), followed by 2294 regulation relations, 2131 positive regulation, and 1920 negative regulation. Supplementary Section 4 has annotation statistics for all relation types and Supplementary Section 5 has the distribution of relations in the training, development, and test sets. Since there are four different biomedical NE types annotated in the corpus, the number of relations grouped by these types is presented in Table 1. RegulaTome offers a vast and varied set of relations for training neural network models for multi-label RE. More than 95% of these relations occur within sentences, while the remaining relations span across sentences. The corpus also features a significant number of NEs, with 38 931 Protein, 4703 Chemical, 3839 Complex, and 7478 Family, summing to 54 951 entities for all entity types.

**Table 1.** Number of annotated relations between the different NE types in RegulaTome

NE types	Relation count
Protein–protein	10 593
Protein–family	2320
Protein–complex	1703
Protein–chemical	1310
Family–family	339
Family–chemical	228
Family–complex	219
Complex–chemical	146
Complex–complex	86
Chemical–chemical	17

### RE system evaluation

We used an extended grid search to find the optimal values of hyper-parameters on the development set of the RegulaTome corpus. Our best result was achieved using the RoBERTa-large-PM-M3-Voc model [5] and the following set of hyper-parameters: “MSL = 128, learning rate = 4e-6, training epochs = 26, and batch size = 16.”

Our best experiment achieved an average precision of 68.9%, an average recall of 67.0%, and an average *F*1-score of 67.9% on the RegulaTome development set. The four models used in this experiment and the evaluation scores measured on the development set are shown in Table 2.

**Table 2.** Performance of the best experiment on the RegulaTome development set

	Precision	Recall	*F*1-score
**Model-1**	**69.1**	**67.4**	**68.3**
Model-2	68.3	66.3	67.3
Model-3	69.7	66.8	68.2
Model-4	68.6	67.3	67.9
Average	68.9	67.0	67.9
SD	0.61	0.51	0.45

The best model (highlighted in bold) is used to perform a run on the held-out test set and for a large-scale run on the entire biomedical scientific literature.

The best model presented in Table 2 (model-1) achieved 66.6% *F*1-score (67.7% precision, 65.5% recall) on the RegulaTome held-out test set.

In Supplementary Section 6, evaluation metrics on the test set are presented on a per-relation-label basis. Complex formation—the label with the highest level of support—is, unsurprisingly, among the relations where the model achieves its best performance (*F*1- score = 78.8%). Performance varies significantly for the catalysis of posttranslational modification relations, with *F*1-scores varying from 85.7% for catalysis of deubiquitination to 0% for another catalysis of small protein removal. Results in these cases seem to be directly affected by the level of support per label (Supplementary Section 4), with labels with a higher level of support, such as catalysis of ubiquitination, catalysis of phosphorylation, catalysis of dephosphorylation, and catalysis of methylation, having *F*1-scores ∼70%. Regulation-related labels seem to be the most difficult to predict, a result consistent with the literature on similar tasks [8]. Relationship sign assignment seems to be easier than the general class prediction, with positive regulation and negative regulation having *F*1-scores >62%, while regulation, despite its high level of support, achieves an *F*1-score of only 49.3%. Moreover, regulation of transcription seems easier to predict than regulation of gene expression, but this could again be explained by the fact that the level of support for regulation of transcription is double that of regulation of gene expression (Supplementary Section 4).

In the next sections, we perform a manual error analysis and a semiautomated label confusion analysis, which allows us to look deeper into these results.

### Manual error analysis

We have selected 20% of documents in the test set and manually analyzed and categorized the errors generated by the best-performing RE model on these documents. An overview of these errors is shown in Table 3, while a case-by-case analysis is provided in Supplementary Section 7.

**Table 3.** Manual error analysis on 20% of documents in the RegulaTome test set

	Count		
Error type	FP	FN	Total
Ambiguous keyword	65	35	100
Rare keyword	0	57	57
Co-reference resolution	31	29	60
Convoluted text excerpt	61	55	116
Model error	17	32	49
Annotation error	26	15	41
Total	200	223	423

From the error categories presented in Table 3, the main sources of errors appear to be “ambiguous keyword” and “convoluted text excerpt,” with over half of the errors being a result of these. The first category encapsulates instances where ambiguous words, such as “target,” can denote either a regulatory (e.g. “The promoter of the CD19 gene is a ‘target’ for BSAP”) relation or a catalytic (e.g. “Tea1 is a substrate ‘target’ of Shk1”) relation and result in model confusion. The second most common category (“convoluted text excerpt”) encompasses text segments with complex syntax, including intricate sentences and cross-sentence relations, which are inherently difficult to annotate and subsequently predict. A closely related category is “coreference resolution,” where the syntactical structure makes it especially difficult for the model to determine the subject to which a given relation pertains, resulting in both false positives (FPs) and false negatives (FNs). The “rare keyword” category results only in FNs as a consequence of words or phrases rarely found in scientific texts (e.g. “protection from inhibition or non-covalent association”), which are recognized and correctly annotated by biology experts, but do not result in enough examples for the model to train on to have a chance to detect them during prediction.

There are two more categories—with lower numbers of errors—which are inherently different than the rest of the categories presented earlier. “Model error” refers to cases where there are clear keywords to denote relations and where there were no clear explanations as to why these have not been correctly predicted by the model. On the other hand, “annotation error” refers to cases in which annotators have inaccurately labeled or not labeled relations, frequently as a result of text ambiguity, which would require correction in the corpus.

### Label confusion analysis

Next, we have categorized the errors based on the confusion of relation labels (Supplementary Sections 8 and 9). Overall, the vast majority of all FPs (81%) are cases where relations are predicted and there should be no relation of any type according to our manual annotations (Supplementary Section 8, bold and italics). Similarly, 82% of FNs are relations that were completely missed (Supplementary Section 9, bold and italics) and are not a result of confusion between labels predicted by the model. For a full categorization of each FP and FN in the RegulaTome test set in terms of label confusion, refer to the Supplementary Table (“Error analysis full results”) available via Zenodo.

For the remaining errors, some label confusion categories are less severe than others. Specifically, 10% of all errors in the test set (126 out of 1048 FPs and 118 out of 1160 FNs)—i.e. half of the remaining errors—have to do with confusion among closely related labels (“Supplementary Sections 8 and 9”). For example, in the regulation of gene expression branch (Fig. 1), either a too-specific label (i.e. regulation of transcription instead of regulation of gene expression) or a too-broad label (i.e. regulation of gene expression instead of regulation of transcription) was predicted. If all confusion within the regulation of gene expression branch was ignored, i.e. if all confusion between regulation of transcription, regulation of translation, and regulation of gene expression labels is counted as true positives (TPs) instead of FPs and FNs, the average *F*1-score for the regulation of gene expression branch increases to 68.8%, which is 9% better than regulation of transcription and 15% better than regulation of gene expression (Supplementary Section 6). Similarly, if all confusion within the catalysis of posttranslational modification branch is ignored, the average *F*1-score for catalysis of posttranslational modification increases to 70.6%, which is better than the *F*1-scores for 18 of the 22 relation types within that branch (Supplementary Section 6).

### Error analysis of direction and sign

The directed relations that can be mined from the literature using our model can provide important information for the analysis of regulatory networks. In this use case, relations are viewed as edges, and what matters most is to have the correct edges, with the right direction, and ideally the right sign (i.e. positive regulation or negative regulation). To evaluate the usefulness of our model’s predictions for this purpose, we categorized label confusion errors into six categories, considering only directed predictions and annotations (i.e. the presence or absence of predicted or annotated complex formation has no impact), namely cases where the model

1. failed to assign a directed interaction, where there should be one,
2. assigned a directed interaction, where there should be none,
3. assigned a directed interaction, but the direction is wrong,
4. failed to assign a sign (positive or negative), where there should be one,
5. assigned a sign, where there should be none, and
6. assigned a sign, but the sign is wrong.

We found 1394 edges with correctly assigned directions and 737, 620, and 5 errors from the first three categories, respectively. While the network that would be produced is somewhat incomplete—missing 737 interactions—its precision would be 70% in terms of connecting the right entities with an edge pointing the right way. It should be noted that in reality, the precision would be even higher since some of the relations counted as FPs are annotation errors in the corpus. Of the correctly detected directed edges, 539 have the correct sign, 31 are missing a sign (Category 4), and 61 have a wrong sign (47 from Category 5 and 14 from Category 6). For the remaining 763 edges, we correctly did not predict a sign. These results further showcase the potential of deep learning–based models trained on RegulaTome for downstream biomedical applications. For details on calculations presented in this section, refer to Supplementary Section 10.

### Large-scale execution for protein relations

We used the best model to extract relations from >36 million PubMed abstracts (as of March 2024) and 6 million articles from the PMC BioC open access collection [29] (as of November 2023). The Jensenlab tagger [30] was used to obtain matches for Protein NEs with normalizations to Ensembl [31] identifiers, and the results were filtered to documents that contain at least two NEs and as a result at least one pair for prediction. A total of 6 920 139 documents complied with this criterion (3 157 239 abstracts and full-text and 3 762 900 abstracts only), which were converted to BRAT standoff format and provided to the model for relation prediction. Predictions were produced for >1.2 billion pairs, with ∼1.5% (18.4 million) having at least one “positive” label. A tab-delimited file with results from the large-scale run is provided through Zenodo.

## Conclusions

In this work, we introduced RegulaTome, a corpus aimed at enhancing biomedical RE, with a focus on proteins, protein-containing entities such as complexes and families, and chemicals. This work represents a significant advancement in the field of biomedical text mining, addressing a limitation of several existing RE corpora that mainly focus on single-type relations at the sentence level. RegulaTome distinguishes itself by its breadth, encompassing 2521 documents with 16 961 relations between 54 951 entities. It is meticulously curated to include 43 types of relations, extending well beyond the scope traditionally covered in biomedical RE tasks, thereby establishing a new standard for complexity and depth in the field.

The effectiveness of RegulaTome is further demonstrated through the deployment of a transformer-based model, which has shown remarkable accuracy in RE, achieving an *F*1-score of 66.6% that underlines the corpus’s utility in accurately identifying and categorizing a diverse range of biological relations. This achievement showcases the corpus’s capacity to broaden the scope of detectable relations and its potential to significantly enhance the development of sophisticated, efficient, and accurate RE systems for biomedical applications. By providing RegulaTome to the scientific community, we aim to facilitate the advancement of biomedical RE systems both through theoretical research and practical applications in the field. Our work sets a new benchmark in biomedical text mining and opens up new avenues for exploring and validating a plethora of complex relations between biomedical entities.

## Supplementary Material

baae095_Supp

## Data Availability

Data underlying this article are available in its online supplementary material and are openly accessible via Zenodo (https://zenodo.org/doi/10.5281/zenodo.10808330) and GitHub (https://github.com/farmeh/RegulaTome_extraction).

## References

[1] Milosevic N , Thielemann W. Comparison of biomedical relationship extraction methods and models for knowledge graph creation. *J Web Semant* 2023;75:100756.

[2] Szklarczyk D , Kirsch R, Koutrouli M et al. The string database in 2023: protein–protein association networks and functional enrichment analyses for any sequenced genome of interest. *Nucleic Acids Res* 2023;51:D638–46. 36370105 10.1093/nar/gkac1000 PMC9825434

[3] Lee K , Lee S, Park S et al. Bronco: biomedical entity relation oncology corpus for extracting gene-variant-disease-drug relations. *Database* 2016;2016:baw043. 10.1093/database/baw043 PMC4830473 27074804

[4] Lee J , Yoon W, Kim S et al. BioBERT: a pre-trained biomedical language representation model for biomedical text mining. *Bioinformatics* 2019;36:1234–40. 10.1093/bioinformatics/btz682 PMC7703786 31501885

[5] Lewis P , Ott M, Du J et al. Pretrained language models for biomedical and clinical tasks: understanding and extending the state-of-the-art. In: *Proceedings of the 3rd Clinical Natural Language Processing Workshop, Association for Computational Linguistics*, Online. pp. 146–57, 2020.

[6] Bunescu R , Ge R, Kate RJ et al. Comparative experiments on learning information extractors for proteins and their interactions. *Artif Intell Med* 2005;33:139–55. 15811782 10.1016/j.artmed.2004.07.016

[7] Herrero-Zazo M , Segura-Bedmar I, Martínez P et al. The DDI corpus: an annotated corpus with pharmacological substances and drug–drug interactions. *J Biomed Informat* 2013;46:914–20. 10.1016/j.jbi.2013.07.011 23906817

[8] Miranda-Escalada A , Mehryary F, Luoma J et al. Overview of DrugProt task at BioCreative VII: data and methods for large-scale text mining and knowledge graph generation of heterogenous chemical–protein relations. *Database* 2023;2023:baad080. 10.1093/database/baad080 PMC10683943 38015956

[9] Pyysalo S , Ginter F, Heimonen J et al. Bioinfer: a corpus for information extraction in the biomedical domain. *BMC Bioinf* 2007;8:1–24. 10.1186/1471-2105-8-50 PMC1808065 17291334

[10] Krallinger M , Leitner F, Rodriguez-Penagos C et al. Overview of the protein-protein interaction annotation extraction task of BioCreative II. *Genome Biol* 2008;9:1–19. 10.1186/gb-2008-9-s2-s4 PMC2559988 18834495

[11] Li J , Sun Y, Johnson RJ et al. BioCreative V CDR task corpus: a resource for chemical disease relation extraction. *Database* 2016;2016:1-10. 10.1093/database/baw068 PMC4860626 27161011

[12] Doughty E , Kertesz-Farkas A, Bodenreider O et al. Toward an automatic method for extracting cancer- and other disease-related point mutations from the biomedical literature. *Bioinformatics* 2011;27:408–15. 21138947 10.1093/bioinformatics/btq667 PMC3031038

[13] Luo L , Lai P-T, Wei C-H et al. BioRED: a rich biomedical relation extraction dataset. *Brief Bioinf* 2022;23:bbac282. 10.1093/bib/bbac282 PMC9487702 35849818

[14] Su J , Wu Y, Ting H-F et al. Renet2: high-performance full-text gene–disease relation extraction with iterative training data expansion. *NAR Genomics Bioinform* 2021;3:lqab062. 10.1093/nargab/lqab062 PMC8256824 34235433

[15] Kim J-D , Ohta T, Pyysalo S et al. Overview of BioNLP’09 shared task on event extraction. In *Proceedings of the BioNLP 2009 Workshop Companion Volume for Shared Task. Association for Computational Linguistics*, Boulder, Colorado. pp. 1–9, 2009.

[16] Ohta T , Pyysalo S, Miwa M et al. Event extraction for post-translational modifications. In: *Proceedings of the 2010 Workshop on Biomedical Natural Language Processing*, Uppsala, Sweden. pp. 19–27, Association for Computational Linguistics, 2010.

[17] Pyysalo S , Ohta T, Rak R et al. Overview of the ID, EPI And REL tasks of BioNLP shared task 2011. *BMC Bioinf* 2012;13:1–26. 10.1186/1471-2105-13-S11-S2 PMC3384257 22759456

[18] Aleksander SA , Balhoff J, Carbon S et al. The gene ontology knowledgebase in 2023. *Genetics* 2023;224:iyad031. 10.1093/genetics/iyad031 PMC10158837 36866529

[19] Ashburner M , Ball CA, Blake JA et al. Gene ontology: tool for the unification of biology. *Nat Genet* 2000;25:25–29. 10802651 10.1038/75556 PMC3037419

[20] Mehryary F , Nastou K, Ohta T et al. String-ing together protein complexes: extracting physical protein interactions from the literature. BioRxiv, 2023. doi: 10.1101/2023.12.10.570999. PMC11441320 39276156

[21] Oughtred R , Rust J, Chang C et al. The BioGRID database: a comprehensive biomedical resource of curated protein, genetic, and chemical interactions. *Protein Sci* 2021;30:187–200. 33070389 10.1002/pro.3978 PMC7737760

[22] Orchard S , Ammari M, Aranda B et al. The MIntACT project—intact as a common curation platform for 11 molecular interaction databases. *Nucleic Acids Res* 2014;42:D358–63. 24234451 10.1093/nar/gkt1115 PMC3965093

[23] Licata L , Briganti L, Peluso D et al. MINT, the molecular interaction database: 2012 update. *Nucleic Acids Res* 2012;40:D857–61. 22096227 10.1093/nar/gkr930 PMC3244991

[24] Gillespie M , Jassal B, Stephan R et al. The reactome pathway knowledgebase 2022. *Nucleic Acids Res* 2022;50:D687–92. 34788843 10.1093/nar/gkab1028 PMC8689983

[25] Paysan-Lafosse T , Blum M, Chuguransky S et al. InterPro in 2022. *Nucleic Acids Res* 2023;51:D418–27. 36350672 10.1093/nar/gkac993 PMC9825450

[26] Stenetorp P , Pyysalo S, Topi´c G et al. brat: a web-based tool for NLP-assisted text annotation. In: *Proceedings of the Demonstrations at the 13th Conference of the European Chapter of the Association for Computational Linguistics*, Avignon, France. pp. 102–07, Association for Computational Linguistics, 2012.

[27] Mehryary F , Björne J, Pyysalo S et al. Deep learning with minimal training data: TurkuNLP entry in the BioNLP shared task 2016. In: *Proceedings of the 4th BioNLP Shared Task Workshop*, Berlin, Germany. pp. 73–81, 2016.

[28] Smith L , Tanabe LK, Ando RJN et al. Overview of BioCreative II gene mention recognition. *Genome Biol* 2008;9:1–19. 10.1186/gb-2008-9-s2-s2 PMC2559986 18834493

[29] Comeau DC , Wei C-H, Islamaj Do˘gan R et al. PMC text mining subset in BioC: about three million full-text articles and growing. *Bioinformatics* 2019;35:3533–35. 30715220 10.1093/bioinformatics/btz070 PMC6748740

[30] Jensen LJ . One tagger, many uses: illustrating the power of ontologies in dictionary-based named entity recognition. bioRxiv 2016:067132.

[31] Martin FJ , Amode MR, Aneja A et al. Ensembl 2023. *Nucleic Acids Res* 2022;51:D933–41. 10.1093/nar/gkac958 PMC9825606 36318249

