# Supplementary material for: RegulaTome: a corpus of typed, directed, and signed relations between biomedical entities in the scientific literature
Source: Database (Oxford). 2024 Sep 12;2024:baae095. doi: 10.1093/database/baae095 (PMC11394941; doi:10.1093/database/baae095)
Supplement: baae095_Supp [file baae095_supp.zip › suppl_data/Supplementary Material - RegulaTome-1.pdf]

Supplementary material

“*RegulaTome: a corpus of typed, directed, and signed relations between biomedical entities in the scientific literature*”

Katerina Nastou[1,†], Farrokh Mehryary[2,†], Tomoko Ohta[3], Jouni Luoma[2], Sampo Pyysalo[2] and Lars Juhl Jensen[1]

[1]Novo Nordisk Foundation Center for Protein Research, University of Copenhagen, Blegdamsvej 3, 2200, Copenhagen, Denmark, [2]TurkuNLP Group, Department of Computing, University of Turku, Turku, Finland, [3]Textimi, Tokyo, Japan

[†] Equal contribution

Supplementary Section 1: Relationship types in RegulaTome

Relationship Type	GO ID	Parent Term
Complex formation	GO:0065003	Relationship
Regulation	GO:0065009	Relationship
Positive regulation	GO:0044093	Regulation
Negative regulation	GO:0044092	Regulation
Regulation of gene expression	GO:0010468	Relationship
Regulation of transcription	GO:0006355	Regulation of gene expression
Regulation of translation	GO:0006417	Regulation of gene expression
Regulation of degradation	GO:0009894	Relationship
Catalysis of post-translational modification	GO:0043687	Relationship
Catalysis of small protein conjugation/removal	GO:0070647	Catalysis of post-translational modification
Catalysis of small protein conjugation	GO:0032446	Catalysis of small protein conjugation/removal
Catalysis of ubiquitination	GO:0016567	Catalysis of small protein conjugation
Catalysis of SUMOylation	GO:0016925	Catalysis of small protein conjugation
Catalysis of neddylation	GO:0045116	Catalysis of small protein conjugation
Other catalysis of small protein conjugation	---	Catalysis of small protein conjugation
Catalysis of small protein removal	GO:0070646	Catalysis of small protein conjugation/removal
Catalysis of deubiquitination	GO:0016579	Catalysis of small protein removal
Catalysis of deSUMOylation	GO:0016926	Catalysis of small protein removal
Catalysis of deneddylation	GO:0000338	Catalysis of small protein removal
Other catalysis of small protein removal	---	Catalysis of small protein removal
Catalysis of phosphoryl group conjugation/removal	---	Catalysis of post-translational modification
Catalysis of phosphorylation	GO:0006468	Catalysis of phosphoryl group conjugation/removal
Catalysis of dephosphorylation	GO:0006470	Catalysis of phosphoryl group conjugation/removal
Catalysis of small molecule conjugation/removal (excluding phosphoryl group)	---	Catalysis of post-translational modification
Catalysis of small molecule conjugation	---	Catalysis of small molecule conjugation/removal (excluding phosphoryl group)
Catalysis of methylation	GO:0006479	Catalysis of small molecule conjugation
Catalysis of acylation	GO:0043543	Catalysis of small molecule conjugation

Relationship Type	GO ID	Parent Term
Catalysis of acetylation	GO:0006473	Catalysis of acylation
Catalysis of palmitoylation	GO:0018345	Catalysis of acylation
Catalysis of lipidation	GO:0006497	Catalysis of small molecule conjugation
Catalysis of prenylation	GO:0018342	Catalysis of lipidation
Catalysis of farnesylation	GO:0018343	Catalysis of prenylation
Catalysis of geranylgeranylation	GO:0018344	Catalysis of prenylation
Catalysis of ADP-ribosylation	GO:0070212	Catalysis of small molecule conjugation
Catalysis of glycosylation	GO:0006486	Catalysis of small molecule conjugation
Other catalysis of small molecule conjugation	---	Catalysis of small molecule conjugation
Catalysis of small molecule removal	---	Catalysis of small molecule conjugation/removal (excluding phosphoryl group)
Catalysis of demethylation	GO:0006482	Catalysis of small molecule removal
Catalysis of deacylation	GO:0035601	Catalysis of small molecule removal
Catalysis of deacetylation	GO:0006476	Catalysis of deacylation
Catalysis of depalmitoylation	GO:0002084	Catalysis of deacylation
Catalysis of deglycosylation	GO:0006517	Catalysis of small molecule removal
Other catalysis of small molecule removal	---	Catalysis of small molecule removal

Supplementary Section 2: Document selection for PTM triage set

Document selection for the construction of the PTM triage set involved five steps in total (`PTM triage 01` to `PTM triage 05`), all of which follow a similar logic, adapted based on relationship counts after each annotation round. The purpose of this expansion is to enrich the corpus in `Catalysis of protein modification` relationships, excluding `Catalysis of phosphorylation` for which we already had high support in the corpus.

PTM triage 01

To generate `PTM triage 01` we searched **UniProtKB** (accessed on January 24th, 2023) using the query `"(GO:0036211) AND (cc_catalytic_activity:*) NOT (GO:0016301) NOT (GO:0006468)"` to identify entries associated with the GO term `protein modification process` while excluding entries related to `protein kinase` and `protein phosphorylation`. This search yielded a total of 1,389,360 results, of which 12,522 were sourced from SwissProt and 1,376,838 from TrEMBL. Subsequently, files from the **Reactome** database were utilized to map the UniProt identifiers from the search above to Reactome reactions and to obtain associated PubMed identifiers (PMIDs) used to describe these reactions. Specifically, the `UniProt2Reactome_PE_Reactions.txt` file was used to map UniProt identifiers to Reactome pathway identifiers, and the `Reaction_PMIDs.txt` file provided information on research publications associated with these Reactome pathways. Reactions containing proteins from the initial list were extracted, resulting in 12,908 unique reactions, which linked to 5,548 unique PMIDs from research papers used to annotate them in Reactome. We then run an NER system on these abstracts, to identify those containing 2-40 entities (to be consistent with all selection steps in ComplexTome), which reduced the number of documents to 4,771. Afterwards we checked which of these documents we have already annotated (97 documents in total), and by removing those documents we are left with the final set of **4,674** documents. We converted these documents to JSON format, shuffled them to be in random order and set up a **prodigy** server to contain all the documents. This would allow us to manually inspect the documents to identify those that contain relationships of interest. This step was deemed necessary, as our previous effort to focus on documents containing `Catalysis of protein modification` relationships, by annotating documents in existing corpora (`Exhaustive PTM corpus`) resulted in very low density of the relationship types we were aiming for. For that reason we decided to "***triage***" the documents, by manually inspecting them and selecting those that contain the relationships of interest. After inspecting 850 documents we selected 100 of them,on the basis of mentioning a `catalysis of protein modification` relationship, besides `catalysis of phosphorylation`. These documents were later annotated and constitute the `PTM triage 01` set in our corpus.

PTM triage 02

For `PTM triage 02` we decided to focus on specific relationship types that were even more underrepresented in the corpus. We followed a similar document selection strategy, but this time we searched UniProt (accessed on March 4th, 2023) for entries that have a field of `catalytic activity` and a GO term for `Catalysis of methylation (GO:0006479)` or `Catalysis of acylation (GO:0043543)` or `Catalysis of lipidation (GO:0006497)` or `Catalysis of Glycosylation (GO:0006486)` or `Catalysis of Demethylation (GO:0006482)` or `Catalysis of Deacylation (GO:0035601)` which includes `Catalysis of deacetylation` and `Catalysis of depalmitoylation` or `Catalysis of Deglycosylation (GO:0006517)`. This search resulted in 380,014 entries; 4,094 from SwissProt and 375,920 from TrEMBL. We then searched Reactome for papers used to annotate reactions with these enzymes and filtered the resulting list to remove papers we already have in the corpus, or seen in the previous triage round and rejected. We also applied an extra filter on top to keep only papers with that contain at least on of the following lemmas (case insensitive) `acetyl|Acyl|add|ADPribosyl|ADP-ribosyl|attach|biotinyl|carbonyl|carboxyl|conjuga|Deacetyl|Deacyl|deconjug|Deglycosyl|deiminat|Demethyl|Deneddyl|Depalmitoyl|Dephosphoryl|DeSUMOyl|Deubiquitin|farnesyl|Gammacarboxyl|gamma-carboxyl|geranylgeranyl|GlcNAcyl|glycoform|Glycosyl|incorporat|isoprenyl|link|lipidmodif|lipidat|lipoyl|methyl|modif|monoacyl|monoprenyl|monoubiquitin|myristol|myristyl|N-glycosyl|neddyl|O-GlcNAcyl|O-glycosyl|O-link|O-Sulf|oxidat|Palmitoyl|Prenyl|remov|repalmitoylat|sulf|SUMO|ubiquitin`. This resulted in a set of 1,046 papers, which were again shuffled and added on prodigy for triage. After checking 428 of these abstracts, we selected 100 of them on the basis of containing the subset of `Catalysis of protein modification` relationships on which our UniProt search is also focused on, and created the `PTM triage 02` set.

PTM triage 03

Similarly to the creation we focused on specific relationship types during this round. Specifically, we focused on relationships that had a total support of less than 100 relationships in the annotated corpus up to that point and we searched Uniprot on March 28th, 2023 for entries that have a field of `"catalytic activity"` and a GO term for `Catalysis of ADP-ribosylation (GO:0070212)` or `Catalysis of SUMOylation (GO:0016925)` or `Catalysis of Acetylation (GO:0006473)` or `Catalysis of Neddylation (GO:0045116)` or `Catalysis of farnesylation (GO:0018343)` or `Catalysis of geranylgeranylation (GO:0018344)` or `Catalysis of Myristoylation (GO:0018377)` or `Catalysis of Glycosylation (GO:0006486)` or `Catalysis of Palmitoylation (GO:0018345)` or `Catalysis of Deubiquitination (GO:0016579)` or `Catalysis of DeSUMOylation (GO:0016926)` or `Catalysis of Deneddylation (GO:0000338)` or `Catalysis of`

`Dephosphorylation (GO:0006470)` or `Catalysis of Demethylation (GO:0006482)` or `Catalysis of Deacetylation (GO:0006476)` or `Catalysis of Depalmitoylation (GO:0002084)` or `Catalysis of Deglycosylation (GO:0006517)`. This search resulted in 431,963 entries; 4,473 from SwissProt and 427,490 from TrEMBL. We applied the exact same strategy to select documents from Reactome as for `PTM triage 02`, and ended up with a set of 1,229 papers, out of which 100 were selected if they contained any of the relationships mentioned above.

PTM triage 04

Once again the focus was on specific relationship types. In this selection round we searched Uniprot on May 8th, 2023 for entries with a field of `"catalytic activity"` and a GO term for `Catalysis of small protein conjugation (GO:0032446)` or `Catalysis of small protein removal (GO:0070646)` or `Catalysis of Dephosphorylation (GO:0006470)` or `Catalysis of Demethylation (GO:0006482)` or `Catalysis of Deacetylation (GO:0006476)` or `Catalysis of Depalmitoylation (GO:0002084)` or `Catalysis of Deglycosylation (GO:0006517)`. This search resulted in 487,244 entries; 4,478 from SwissProt and 482,766 from TrEMBL. We applied the exact same strategy to select documents from Reactome as for `PTM triage 02` and `03`, and once more selected 100 documents out of 900 if they contained any of the relationships mentioned above to generate `PTM triage 04`.

PTM triage 05

For `PTM triage 05` we continued using the same documents as for PTM triage 04, but ignored documents mentioning `Catalysis of small protein removal (GO:0070646)` during triage. We selected 100 more documents this way and thus concluded all the selection steps for the `PTM triage` set.

Supplementary Section 3: Document selection for Reactome full-text excerpts set

For ComplexTome we had already selected 300 abstracts extracted from 21,941 papers used for pathway annotation in the Reactome pathway knowledgebase. We used the same pool of papers to select 500 full-text paragraphs for the `Reactome full-text excerpts` set. We split these documents in paragraphs and ended up with a set of 384,696 total paragraphs. Then we used the [codebase]() presented in the S1000 paper (*Luoma, et al, 2023*) to train a transformer-based model for NER of all named entity types annotated in the RegulaTome corpus, i.e. `Protein`, `Complex`, `Protein Family` and `Chemical`. We used the same document split we already have to generate a training, development and test set, and then used the named entity annotations to train a transformer-based model for multi-class classification of named entities. We used the best model based on a `RoBERTa-large-PM-M3-Voc` model and then predicted named entities in all the documents above. We shuffled the paragraphs and then used the following criteria to filter down the entire pool of paragraphs from which we would then select documents for each step of generating the `Reactome full-text excerpts` set:

- the paragraphs should contain at least three annotated textbound named entities of type `Protein`, `Complex` or `Protein Family`
- 30% of these names should have not been seen before in the corpus (so selection is done incrementally, one document at a time)
- the paragraph size should be between 50 and 500 words

After selecting documents with these criteria we were left with 61973 paragraphs from which we initially selected 200 documents to generate `Reactome full-text excerpts 01` and `Reactome full-text excerpts 02`. For the next 3 steps we created a custom ChatGPT (available through this link: https://chat.openai.com/g/g-s8Uk5qFoO-biomed-scholar) and instructed it to classify paragraphs from scientific papers into specific sections. We chose this option instead of mapping the paragraphs back to the original documents and identifying the sections, due to differences in how papers are structured in different journals, the lack of need of 100% accuracy in classification and the initial good classification results we were getting with the custom ChatGPT. For batches `Reactome full-text excerpts 03`, `Reactome full-text excerpts 04` and `Reactome full-text excerpts 05`, we filtered documents so that they were either from the Introduction, Results, Discussion or Conclusions sections (according to classification of our custom ChatGPT) and took extra care to manually remove any figure legends from `Reactome full-text excerpts 04` and `Reactome full-text excerpts 05` sets. We did that as we observed that paragraphs from sections other than those mentioned above mostly did not contain any of the relationships we annotate in this corpus, while we were annotating the first two batches of this set.

Supplementary Section 4: Relationship statistics for RegulaTome

Relationship type	Relationship count
Complex formation	6463
Regulation	2294
Positive regulation	2130
Negative regulation	1920
Regulation of gene expression	521
Regulation of transcription	899
Regulation of translation	21
Regulation of degradation	336
Catalysis of posttranslational modification	169
Catalysis of small protein conjugation or removal	5
Catalysis of small protein conjugation	42
Catalysis of ubiquitination	474
Catalysis of SUMOylation	86
Catalysis of neddylation	20
Other catalysis of small protein conjugation	4
Catalysis of small protein removal	1
Catalysis of deubiquitination	79
Catalysis of deSUMOylation	11
Catalysis of deneddylation	19
Other catalysis of small protein removal	3
Catalysis of phosphoryl group conjugation or removal	7
Catalysis of phosphorylation	442
Catalysis of dephosphorylation	213
Catalysis of other small molecule conjugation or removal	9
Catalysis of small molecule conjugation	0
Catalysis of methylation	259
Catalysis of glycosylation	43
Catalysis of ADP-ribosylation	31
Catalysis of acylation	14
Catalysis of acetylation	129
Catalysis of palmitoylation	66
Catalysis of lipidation	3
Catalysis of prenylation	1
Catalysis of farnesylation	3
Catalysis of geranylgeranylation	17
Other catalysis of small molecule conjugation	33
Catalysis of small molecule removal	2
Catalysis of demethylation	110
Catalysis of deglycosylation	1
Catalysis of deacylation	6
Catalysis of deacetylation	53
Catalysis of depalmitoylation	6
Other catalysis of small molecule removal	16

Supplementary Section 5: Relationship statistics in the training, development and test sets

Relationship type	# Train	# Devel	# Test	% Train	% Devel	%Test
Complex formation	3984	1258	1221	61.6%	19.5%	18.9%
Regulation	1330	480	484	58.0%	20.9%	21.1%
Positive regulation	1272	415	443	59.7%	19.5%	20.8%
Negative regulation	1113	390	417	58.0%	20.3%	21.7%
Regulation of gene expression	319	105	97	61.2%	20.2%	18.6%
Regulation of transcription	559	168	172	62.2%	18.7%	19.1%
Regulation of translation	8	6	7	38.1%	28.6%	33.3%
Regulation of degradation	201	86	49	59.8%	25.6%	14.6%
Catalysis of posttranslational modification	94	31	44	55.6%	18.3%	26.0%
Catalysis of small protein conjugation or removal	3	0	2	60.0%	0.0%	40.0%
Catalysis of small protein conjugation	29	8	5	69.0%	19.0%	11.9%
Catalysis of ubiquitination	289	91	94	61.0%	19.2%	19.8%
Catalysis of SUMOylation	57	15	14	66.3%	17.4%	16.3%
Catalysis of neddylation	13	5	2	65.0%	25.0%	10.0%
Other catalysis of small protein conjugation	3	0	1	75.0%	0.0%	25.0%
Catalysis of small protein removal	1	0	0	100.0%	0.0%	0.0%
Catalysis of deubiquitination	49	15	15	62.0%	19.0%	19.0%
Catalysis of deSUMOylation	7	4	0	63.6%	36.4%	0.0%
Catalysis of deneddylation	11	2	6	57.9%	10.5%	31.6%
Other catalysis of small protein removal	2	0	1	66.7%	0.0%	33.3%
Catalysis of phosphoryl group conjugation or removal	6	0	1	85.7%	0.0%	14.3%
Catalysis of phosphorylation	252	91	99	57.0%	20.6%	22.4%
Catalysis of dephosphorylation	126	42	45	59.2%	19.7%	21.1%
Catalysis of other small molecule conjugation or removal	3	0	6	33.3%	0.0%	66.7%

Relationship type	# Train	# Devel	# Test	% Train	% Devel	%Test
Catalysis of methylation	163	45	51	62.9%	17.4%	19.7%
Catalysis of glycosylation	27	8	8	62.8%	18.6%	18.6%
Catalysis of ADP-ribosylation	19	6	6	61.3%	19.4%	19.4%
Catalysis of acylation	8	5	1	57.1%	35.7%	7.1%
Catalysis of acetylation	74	26	29	57.4%	20.2%	22.5%
Catalysis of palmitoylation	42	12	12	63.6%	18.2%	18.2%
Catalysis of lipidation	3	0	0	100.0%	0.0%	0.0%
Catalysis of prenylation	1	0	0	100.0%	0.0%	0.0%
Catalysis of farnesylation	3	0	0	100.0%	0.0%	0.0%
Catalysis of geranylgeranylation	17	0	0	100.0%	0.0%	0.0%
Other catalysis of small molecule conjugation	27	2	4	81.8%	6.1%	12.1%
Catalysis of small molecule removal	2	0	0	100.0%	0.0%	0.0%
Catalysis of demethylation	73	19	18	66.4%	17.3%	16.4%
Catalysis of deglycosylation	1	0	0	100.0%	0.0%	0.0%
Catalysis of deacylation	3	3	0	50.0%	50.0%	0.0%
Catalysis of deacetylation	35	9	9	66.0%	17.0%	17.0%
Catalysis of depalmitoylation	5	1	0	83.3%	16.7%	0.0%
Other catalysis of small molecule removal	2	14	0	12.5%	87.5%	0.0%

Supplementary Section 6: Evaluation statistics per class on the RegulaTome held-out test set

TP: True Positive, FN: False Negative, FP: False Positive

Relationship	Precision	TP/TP+FP	Recall	TP/TP+FN	F-score
Catalysis of deubiquitination	92.3%	12/13	80.0%	12/15	85.7%
Complex formation	78.6%	965/1227	79.0%	965/1221	78.8%
Catalysis of demethylation	69.6%	16/23	88.9%	16/18	78.0%
Catalysis of ubiquitination	66.4%	77/116	81.9%	77/94	73.3%
Catalysis of phosphorylation	76.5%	65/85	66.3%	65/98	71.0%
Catalysis of dephosphorylation	68.1%	32/47	71.1%	32/45	69.6%
Catalysis of methylation	70.0%	35/50	68.6%	35/51	69.3%
Catalysis of neddylation	100.0%	1/1	50.0%	1/2	66.7%
Negative regulation	64.8%	263/406	63.2%	263/416	64.0%
Positive regulation	61.7%	276/447	62.6%	276/441	62.2%
Regulation of degradation	51.4%	36/70	73.5%	36/49	60.5%
Regulation of transcription	61.1%	102/167	59.6%	102/171	60.4%
Catalysis of acetylation	60.7%	17/28	58.6%	17/29	59.6%
Catalysis of palmitoylation	58.3%	7/12	58.3%	7/12	58.3%
Regulation of gene expression	59.3%	48/81	49.5%	48/97	53.9%
Catalysis of SUMOylation	58.3%	7/12	50.0%	7/14	53.8%
Catalysis of deacetylation	45.5%	5/11	55.6%	5/9	50.0%
Regulation	53.0%	223/421	46.1%	223/484	49.3%
Catalysis of ADP-ribosylation	50.0%	2/4	33.3%	2/6	40.0%
Other catalysis of small molecule conjugation	50.0%	1/2	25.0%	1/4	33.3%
Catalysis of small protein conjugation	50.0%	1/2	20.0%	1/5	28.6%
Catalysis of posttranslational modification	37.5%	6/16	13.6%	6/44	20.0%
Catalysis of glycosylation	20.0%	1/5	12.5%	1/8	15.4%
Catalysis of deneddylation	0.0%	0/0	0.0%	0/6	0.0%
Catalysis of other small molecule conjugation or removal	0.0%	0/0	0.0%	0/6	0.0%
Catalysis of phosphoryl group conjugation or removal	0.0%	0/0	0.0%	0/1	0.0%
Catalysis of small protein conjugation or removal	0.0%	0/0	0.0%	0/2	0.0%
Other catalysis of small protein conjugation	0.0%	0/0	0.0%	0/1	0.0%
Other catalysis of small protein removal	0.0%	0/0	0.0%	0/1	0.0%
Regulation of translation	0.0%	0/0	0.0%	0/7	0.0%

Supplementary Section 7: Results of relation extraction manual error analysis results for the best model on 20% of documents on the RegulaTome test set

FN: False Negative, FP: False Positive

PMID	Relationship type	Entity 1	Entity 2	Error type	FP/FN
1939122	Complex formation	T13	T17	ambiguous keyword	FP
1939122	Regulation	T5	T16	ambiguous keyword	FP
9857197	Regulation	T15	T14	ambiguous keyword	FP
9857197	Regulation	T28	T4	ambiguous keyword	FP
9857197	Regulation	T5	T4	ambiguous keyword	FP
10446169	Regulation	T23	T14	ambiguous keyword	FP
10763828	Negative regulation	T13	T15	ambiguous keyword	FP
10763828	Regulation of gene expression	T13	T15	ambiguous keyword	FP
10763828	Regulation of gene expression	T16	T17	ambiguous keyword	FP
10763828	Regulation of transcription	T22	T23	ambiguous keyword	FP
11099047	Complex formation	T18	T20	ambiguous keyword	FP
11099047	Complex formation	T19	T20	ambiguous keyword	FP
14536086	Catalysis of methylation	T14	T12	ambiguous keyword	FP
14536086	Catalysis of methylation	T15	T12	ambiguous keyword	FP
14981544	Regulation of transcription	T27	T28	ambiguous keyword	FP
14981544	Regulation of transcription	T3	T4	ambiguous keyword	FP
15031289	Regulation	T10	T8	ambiguous keyword	FP
15485920	Regulation	T24	T25	ambiguous keyword	FP
15485920	Complex formation	T19	T4	ambiguous keyword	FP
15485920	Regulation	T19	T4	ambiguous keyword	FP
15886116	Regulation	T2	T6	ambiguous keyword	FP
15886116	Regulation	T3	T6	ambiguous keyword	FP
15886116	Regulation	T4	T6	ambiguous keyword	FP
15886116	Regulation	T5	T6	ambiguous keyword	FP
16282325	Regulation	T20	T21	ambiguous keyword	FP
17572495	Regulation of transcription	T2	T3	ambiguous keyword	FP
17572495	Regulation of transcription	T1	T3	ambiguous keyword	FP
17662961	Complex formation	T10	T11	ambiguous keyword	FP
17662961	Complex formation	T11	T12	ambiguous keyword	FP
17948059	Catalysis of methylation	T19	T1	ambiguous keyword	FP
17948059	Catalysis of posttranslational modification	T14	T15	ambiguous keyword	FP
18094119	Regulation of transcription	T19	T18	ambiguous keyword	FP
18158893	Positive regulation	T15	T4	ambiguous keyword	FP
18158893	Negative regulation	T6	T16	ambiguous keyword	FP

PMID	Relationship type	Entity 1	Entity 2	Error type	FP/FN
18794358	Regulation	T13	T14	ambiguous keyword	FP
18794358	Regulation	T13	T15	ambiguous keyword	FP
20368621	Regulation of transcription	T9	T10	ambiguous keyword	FP
21236256	Regulation of transcription	T12	T23	ambiguous keyword	FP
21236256	Regulation of transcription	T12	T24	ambiguous keyword	FP
21236256	Regulation of transcription	T11	T23	ambiguous keyword	FP
21236256	Regulation of transcription	T11	T24	ambiguous keyword	FP
21266407	Complex formation	T10	T11	ambiguous keyword	FP
21266407	Regulation	T16	T4	ambiguous keyword	FP
21554248	Regulation of gene expression	T15	T16	ambiguous keyword	FP
21554248	Regulation of gene expression	T14	T16	ambiguous keyword	FP
21554248	Regulation of gene expression	T13	T16	ambiguous keyword	FP
21554248	Regulation of gene expression	T36	T16	ambiguous keyword	FP
18995841_22	Regulation	T361	T360	ambiguous keyword	FP
18995841_22	Regulation	T373	T374	ambiguous keyword	FP
18995841_22	Regulation	T373	T375	ambiguous keyword	FP
21347277_5	Complex formation	T3	T7	ambiguous keyword	FP
22086907_3	Catalysis of ubiquitination	T8	T13	ambiguous keyword	FP
22958824_7	Complex formation	T8	T9	ambiguous keyword	FP
23247405_12	Regulation	T196	T194	ambiguous keyword	FP
23247405_12	Regulation	T196	T195	ambiguous keyword	FP
23247405_12	Regulation	T197	T194	ambiguous keyword	FP
23247405_12	Regulation	T197	T195	ambiguous keyword	FP
23615448_14	Positive regulation	T251	T248	ambiguous keyword	FP
23615448_14	Positive regulation	T250	T249	ambiguous keyword	FP
25819761_21	Complex formation	T341	T342	ambiguous keyword	FP
32367036_16	Regulation	T196	T195	ambiguous keyword	FP
9412462_6	Regulation	T68	T69	ambiguous keyword	FP
9412462_6	Regulation	T68	T71	ambiguous keyword	FP
9412462_6	Regulation	T77	T78	ambiguous keyword	FP
9412462_6	Regulation	T77	T79	ambiguous keyword	FP
22086907_3	Catalysis of posttranslational modification	T22	T12	ambiguous keyword	FN
15031289	Catalysis of posttranslational modification	T10	T8	ambiguous keyword	FN
10866691	Complex formation	T17	T16	ambiguous keyword	FN

PMID	Relationship type	Entity 1	Entity 2	Error type	FP/FN
10866691	Complex formation	T20	T19	ambiguous keyword	FN
17108083	Complex formation	T12	T13	ambiguous keyword	FN
18794358	Complex formation	T13	T16	ambiguous keyword	FN
18794358	Complex formation	T13	T17	ambiguous keyword	FN
21236256	Complex formation	T11	T12	ambiguous keyword	FN
23022657_23	Complex formation	T9	T8	ambiguous keyword	FN
23022657_23	Complex formation	T6	T7	ambiguous keyword	FN
9857197	Complex formation	T15	T14	ambiguous keyword	FN
15485920	Complex formation	T24	T25	ambiguous keyword	FN
18794358	Complex formation	T13	T14	ambiguous keyword	FN
18794358	Complex formation	T13	T15	ambiguous keyword	FN
18794358	Positive regulation	T38	T40	ambiguous keyword	FN
18794358	Positive regulation	T38	T41	ambiguous keyword	FN
16282325	Positive regulation	T20	T21	ambiguous keyword	FN
9412462_6	Positive regulation	T68	T69	ambiguous keyword	FN
9412462_6	Positive regulation	T68	T71	ambiguous keyword	FN
9412462_6	Positive regulation	T77	T78	ambiguous keyword	FN
9412462_6	Positive regulation	T77	T79	ambiguous keyword	FN
10763828	Regulation of gene expression	T22	T23	ambiguous keyword	FN
17572495	Regulation of gene expression	T2	T3	ambiguous keyword	FN
17572495	Regulation of gene expression	T1	T3	ambiguous keyword	FN
18158893	Regulation of gene expression	T17	T10	ambiguous keyword	FN
20368621	Regulation of gene expression	T9	T10	ambiguous keyword	FN
18794358	Regulation of transcription	T38	T39	ambiguous keyword	FN
18794358	Regulation of transcription	T38	T40	ambiguous keyword	FN
18794358	Regulation of transcription	T38	T41	ambiguous keyword	FN
21554248	Regulation of transcription	T15	T16	ambiguous keyword	FN
21554248	Regulation of transcription	T14	T16	ambiguous keyword	FN
21554248	Regulation of transcription	T13	T16	ambiguous keyword	FN
21554248	Regulation of transcription	T36	T16	ambiguous keyword	FN
14981544	Regulation	T33	T15	ambiguous keyword	FN
22958824_7	Regulation	T16	T15	ambiguous keyword	FN
1939122	Negative regulation	T18	T7	annotation error	FP
10866691	Catalysis of phosphorylation	T12	T13	annotation error	FP
10866691	Catalysis of phosphorylation	T30	T13	annotation error	FP
10866691	Regulation	T30	T14	annotation error	FP
11983168	Positive regulation	T5	T4	annotation error	FP
12548019	Positive regulation	T2	T1	annotation error	FP
12730668	Negative regulation	T3	T5	annotation error	FP
14536086	Positive regulation	T23	T21	annotation error	FP

PMID	Relationship type	Entity 1	Entity 2	Error type	FP/FN
18794358	Positive regulation	T25	T24	annotation error	FP
18794358	Regulation	T25	T23	annotation error	FP
18794358	Complex formation	T6	T10	annotation error	FP
18794358	Complex formation	T14	T16	annotation error	FP
18794358	Complex formation	T14	T17	annotation error	FP
18794358	Complex formation	T15	T16	annotation error	FP
18794358	Complex formation	T15	T17	annotation error	FP
18794358	Complex formation	T28	T32	annotation error	FP
18794358	Complex formation	T29	T31	annotation error	FP
18794358	Complex formation	T29	T32	annotation error	FP
20457893	Catalysis of demethylation	T1	T2	annotation error	FP
20858899	Negative regulation	T32	T33	annotation error	FP
20858899	Positive regulation	T32	T35	annotation error	FP
21081508	Negative regulation	T6	T8	annotation error	FP
22194618	Positive regulation	T30	T10	annotation error	FP
19637179_9	Complex formation	T19	T20	annotation error	FP
19637179_9	Complex formation	T4	T15	annotation error	FP
22958824_7	Positive regulation	T5	T4	annotation error	FP
21081508	Catalysis of glycosylation	T6	T8	annotation error	FN
17255109	Complex formation	T20	T21	annotation error	FN
18794358	Complex formation	T18	T19	annotation error	FN
20457893	Complex formation	T12	T13	annotation error	FN
29666278	Complex formation	T10	T28	annotation error	FN
18794358	Complex formation	T25	T24	annotation error	FN
18794358	Complex formation	T23	T25	annotation error	FN
15031289	Negative regulation	T17	T19	annotation error	FN
15031289	Negative regulation	T17	T20	annotation error	FN
20858899	Regulation of degradation	T32	T33	annotation error	FN
23240008	Regulation of transcription	T13	T12	annotation error	FN
10770935	Regulation	T14	T10	annotation error	FN
12548019	Regulation	T2	T1	annotation error	FN
20858899	Regulation	T32	T35	annotation error	FN
22958824_7	Regulation	T17	T15	annotation error	FN
14981544	Negative regulation	T36	T30	co-reference resolution	FP
17255109	Negative regulation	T36	T35	co-reference resolution	FP
17255109	Positive regulation	T20	T16	co-reference resolution	FP
17255109	Positive regulation	T21	T16	co-reference resolution	FP
18624796	Complex formation	T3	T7	co-reference resolution	FP
18624796	Complex formation	T4	T5	co-reference resolution	FP
18794358	Catalysis of acetylation	T29	T35	co-reference resolution	FP
18794358	Catalysis of acetylation	T29	T36	co-reference resolution	FP

PMID	Relationship type	Entity 1	Entity 2	Error type	FP/FN
18794358	Complex formation	T26	T27	co-reference resolution	FP
19503082	Regulation	T18	T19	co-reference resolution	FP
20129058	Complex formation	T18	T15	co-reference resolution	FP
20406818	Catalysis of ubiquitination	T6	T8	co-reference resolution	FP
20406818	Complex formation	T25	T27	co-reference resolution	FP
20624928	Positive regulation	T20	T3	co-reference resolution	FP
21236256	Regulation of transcription	T6	T18	co-reference resolution	FP
21236256	Regulation of transcription	T6	T19	co-reference resolution	FP
21236256	Regulation of transcription	T5	T18	co-reference resolution	FP
21236256	Regulation of transcription	T5	T19	co-reference resolution	FP
21266407	Regulation	T11	T23	co-reference resolution	FP
27578797	Regulation	T1	T12	co-reference resolution	FP
29666278	Negative regulation	T3	T13	co-reference resolution	FP
29666278	Regulation of degradation	T3	T13	co-reference resolution	FP
11238463_35	Negative regulation	T272	T269	co-reference resolution	FP
11238463_35	Negative regulation	T272	T270	co-reference resolution	FP
11238463_35	Negative regulation	T273	T270	co-reference resolution	FP
18995841_22	Negative regulation	T371	T370	co-reference resolution	FP
19684574_7	Regulation	T7	T19	co-reference resolution	FP
23022657_23	Complex formation	T6	T10	co-reference resolution	FP
26984517_50	Negative regulation	T311	T314	co-reference resolution	FP
26984517_50	Negative regulation	T311	T315	co-reference resolution	FP
26984517_50	Negative regulation	T312	T314	co-reference resolution	FP
17615152	Catalysis of phosphorylation	T42	T11	co-reference resolution	FN
10446169	Complex formation	T12	T13	co-reference resolution	FN
16282325	Complex formation	T17	T18	co-reference resolution	FN
16282325	Complex formation	T17	T19	co-reference resolution	FN
17255109	Complex formation	T37	T38	co-reference resolution	FN
18794358	Complex formation	T14	T15	co-reference resolution	FN
18794358	Complex formation	T16	T17	co-reference resolution	FN
18794358	Complex formation	T28	T29	co-reference resolution	FN
17615152	Negative regulation	T12	T1	co-reference resolution	FN
17615152	Negative regulation	T13	T1	co-reference resolution	FN
26984517_50	Negative regulation	T310	T314	co-reference resolution	FN
26984517_50	Negative regulation	T310	T315	co-reference resolution	FN
1939122	Positive regulation	T29	T15	co-reference resolution	FN
16282325	Positive regulation	T25	T28	co-reference resolution	FN

PMID	Relationship type	Entity 1	Entity 2	Error type	FP/FN
21554248	Positive regulation	T21	T19	co-reference resolution	FN
21554248	Positive regulation	T35	T42	co-reference resolution	FN
21554248	Positive regulation	T20	T19	co-reference resolution	FN
21554248	Positive regulation	T8	T42	co-reference resolution	FN
17572495	Positive regulation	T30	T29	co-reference resolution	FN
17572495	Positive regulation	T31	T29	co-reference resolution	FN
17572495	Positive regulation	T32	T29	co-reference resolution	FN
17615152	Regulation of gene expression	T12	T1	co-reference resolution	FN
17615152	Regulation of gene expression	T13	T1	co-reference resolution	FN
11983168	Regulation	T19	T22	co-reference resolution	FN
11983168	Regulation	T19	T23	co-reference resolution	FN
14752096	Regulation	T6	T18	co-reference resolution	FN
18094119	Regulation	T9	T6	co-reference resolution	FN
18094119	Regulation	T9	T7	co-reference resolution	FN
27578797	Regulation	T1	T2	co-reference resolution	FN
1939122	Positive regulation	T25	T7	convoluted text excerpt	FP
1939122	Positive regulation	T26	T7	convoluted text excerpt	FP
1939122	Positive regulation	T33	T23	convoluted text excerpt	FP
1939122	Positive regulation	T33	T24	convoluted text excerpt	FP
1939122	Regulation	T30	T15	convoluted text excerpt	FP
2981876	Complex formation	T14	T17	convoluted text excerpt	FP
2981876	Complex formation	T15	T17	convoluted text excerpt	FP
2981876	Complex formation	T16	T17	convoluted text excerpt	FP
2981876	Negative regulation	T23	T8	convoluted text excerpt	FP
2981876	Negative regulation	T23	T9	convoluted text excerpt	FP
10763828	Negative regulation	T26	T25	convoluted text excerpt	FP
10763828	Regulation of gene expression	T26	T25	convoluted text excerpt	FP
10770935	Positive regulation	T18	T7	convoluted text excerpt	FP
10770935	Regulation of transcription	T18	T7	convoluted text excerpt	FP
11099047	Catalysis of deacetylation	T7	T8	convoluted text excerpt	FP
11598127	Catalysis of ubiquitination	T6	T7	convoluted text excerpt	FP
11598127	Positive regulation	T18	T22	convoluted text excerpt	FP
11687605	Negative regulation	T2	T5	convoluted text excerpt	FP
11687605	Regulation of transcription	T2	T5	convoluted text excerpt	FP
11983168	Regulation	T20	T22	convoluted text excerpt	FP
11983168	Regulation	T20	T23	convoluted text excerpt	FP
12730668	Negative regulation	T19	T18	convoluted text excerpt	FP
12730668	Positive regulation	T20	T18	convoluted text excerpt	FP
12730668	Positive regulation	T25	T26	convoluted text excerpt	FP
12730668	Regulation of transcription	T19	T18	convoluted text excerpt	FP
14752096	Catalysis of acetylation	T5	T16	convoluted text excerpt	FP

PMID	Relationship type	Entity 1	Entity 2	Error type	FP/FN
15031289	Negative regulation	T22	T23	convoluted text excerpt	FP
15031289	Negative regulation	T22	T24	convoluted text excerpt	FP
16282325	Regulation	T29	T30	convoluted text excerpt	FP
17108083	Catalysis of ubiquitination	T14	T18	convoluted text excerpt	FP
17108083	Catalysis of ubiquitination	T27	T31	convoluted text excerpt	FP
17255109	Complex formation	T18	T19	convoluted text excerpt	FP
17572495	Positive regulation	T6	T7	convoluted text excerpt	FP
18794358	Complex formation	T6	T11	convoluted text excerpt	FP
18794358	Complex formation	T6	T12	convoluted text excerpt	FP
18794358	Complex formation	T8	T10	convoluted text excerpt	FP
18794358	Complex formation	T8	T11	convoluted text excerpt	FP
18794358	Complex formation	T8	T12	convoluted text excerpt	FP
18794358	Complex formation	T10	T11	convoluted text excerpt	FP
19503082	Positive regulation	T23	T6	convoluted text excerpt	FP
20368621	Negative regulation	T9	T18	convoluted text excerpt	FP
20512148	Positive regulation	T20	T2	convoluted text excerpt	FP
20512148	Regulation of gene expression	T20	T2	convoluted text excerpt	FP
21236256	Positive regulation	T15	T17	convoluted text excerpt	FP
21554248	Regulation of transcription	T11	T42	convoluted text excerpt	FP
21554248	Positive regulation	T23	T24	convoluted text excerpt	FP
21554248	Positive regulation	T27	T38	convoluted text excerpt	FP
22908299	Regulation	T20	T19	convoluted text excerpt	FP
27578797	Negative regulation	T10	T16	convoluted text excerpt	FP
29666278	Catalysis of ubiquitination	T20	T19	convoluted text excerpt	FP
29666278	Catalysis of ubiquitination	T21	T19	convoluted text excerpt	FP
29666278	Catalysis of ubiquitination	T22	T19	convoluted text excerpt	FP
29666278	Regulation of degradation	T20	T19	convoluted text excerpt	FP
29666278	Regulation of degradation	T21	T19	convoluted text excerpt	FP
29666278	Regulation of degradation	T22	T19	convoluted text excerpt	FP
18995841_22	Positive regulation	T354	T357	convoluted text excerpt	FP
18995841_22	Positive regulation	T356	T357	convoluted text excerpt	FP
19637179_9	Regulation	T19	T10	convoluted text excerpt	FP
21951725_19	Complex formation	T230	T237	convoluted text excerpt	FP
22086907_3	Negative regulation	T20	T30	convoluted text excerpt	FP
26984517_50	Negative regulation	T302	T301	convoluted text excerpt	FP
17255109	Catalysis of phosphoryl group conjugation or removal	T1	T2	convoluted text excerpt	FN
19503082	Catalysis of phosphorylation	T24	T9	convoluted text excerpt	FN
23240008	Catalysis of phosphorylation	T9	T10	convoluted text excerpt	FN

PMID	Relationship type	Entity 1	Entity 2	Error type	FP/FN
17018294	Catalysis of SUMOylation	T11	T9	convoluted text excerpt	FN
17255109	Catalysis of ubiquitination	T34	T35	convoluted text excerpt	FN
12730668	Complex formation	T23	T24	convoluted text excerpt	FN
21554248	Complex formation	T11	T9	convoluted text excerpt	FN
21554248	Complex formation	T9	T35	convoluted text excerpt	FN
21554248	Complex formation	T21	T22	convoluted text excerpt	FN
21554248	Complex formation	T20	T22	convoluted text excerpt	FN
32367036_16	Complex formation	T193	T194	convoluted text excerpt	FN
11598127	Negative regulation	T14	T16	convoluted text excerpt	FN
11598127	Negative regulation	T13	T16	convoluted text excerpt	FN
12730668	Negative regulation	T30	T16	convoluted text excerpt	FN
12730668	Negative regulation	T17	T16	convoluted text excerpt	FN
18158893	Negative regulation	T8	T18	convoluted text excerpt	FN
19503082	Negative regulation	T11	T13	convoluted text excerpt	FN
21236256	Negative regulation	T10	T23	convoluted text excerpt	FN
21236256	Negative regulation	T10	T24	convoluted text excerpt	FN
26984517_50	Negative regulation	T303	T302	convoluted text excerpt	FN
32367036_16	Negative regulation	T193	T194	convoluted text excerpt	FN
20512148	Negative regulation	T24	T4	convoluted text excerpt	FN
1939122	Positive regulation	T22	T15	convoluted text excerpt	FN
12548019	Positive regulation	T14	T17	convoluted text excerpt	FN
15031289	Positive regulation	T22	T24	convoluted text excerpt	FN
27578797	Positive regulation	T10	T16	convoluted text excerpt	FN
1939122	Positive regulation	T30	T15	convoluted text excerpt	FN
29666278	Regulation of degradation	T6	T19	convoluted text excerpt	FN
18158893	Regulation of gene expression	T17	T11	convoluted text excerpt	FN
19503082	Regulation of gene expression	T11	T13	convoluted text excerpt	FN
26984517_50	Regulation of gene expression	T303	T302	convoluted text excerpt	FN
20406818	Regulation of transcription	T8	T12	convoluted text excerpt	FN
7524088	Regulation	T4	T7	convoluted text excerpt	FN
9857197	Regulation	T10	T8	convoluted text excerpt	FN
9857197	Regulation	T10	T9	convoluted text excerpt	FN
15886116	Regulation	T28	T13	convoluted text excerpt	FN
17108083	Regulation	T14	T16	convoluted text excerpt	FN
17108083	Regulation	T14	T17	convoluted text excerpt	FN
18094119	Regulation	T17	T14	convoluted text excerpt	FN
18158893	Regulation	T2	T13	convoluted text excerpt	FN
18158893	Regulation	T18	T9	convoluted text excerpt	FN

PMID	Relationship type	Entity 1	Entity 2	Error type	FP/FN
20406818	Regulation	T1	T2	convoluted text excerpt	FN
21236256	Regulation	T1	T16	convoluted text excerpt	FN
21236256	Regulation	T1	T17	convoluted text excerpt	FN
21236256	Regulation	T13	T14	convoluted text excerpt	FN
23240008	Regulation	T20	T19	convoluted text excerpt	FN
29666278	Regulation	T8	T25	convoluted text excerpt	FN
18995841_22	Regulation	T364	T367	convoluted text excerpt	FN
18995841_22	Regulation	T365	T367	convoluted text excerpt	FN
18995841_22	Regulation	T366	T367	convoluted text excerpt	FN
25819761_21	Regulation	T346	T345	convoluted text excerpt	FN
18158893	Regulation	T6	T16	convoluted text excerpt	FN
26984517_50	Regulation	T302	T301	convoluted text excerpt	FN
21554248	Regulation	T23	T24	convoluted text excerpt	FN
21554248	Regulation	T27	T38	convoluted text excerpt	FN
11099047	Catalysis of deacetylation	T12	T13	model error	FP
11099047	Catalysis of deacetylation	T12	T14	model error	FP
11099047	Complex formation	T8	T11	model error	FP
11099047	Catalysis of deacetylation	T20	T21	model error	FP
17662961	Complex formation	T1	T23	model error	FP
18094119	Negative regulation	T9	T7	model error	FP
18158893	Regulation of transcription	T17	T10	model error	FP
20406818	Positive regulation	T16	T17	model error	FP
20512148	Regulation	T24	T4	model error	FP
20858899	Catalysis of small protein conjugation	T36	T37	model error	FP
22194618	Complex formation	T28	T6	model error	FP
21685908_17	Regulation	T11	T10	model error	FP
21951725_19	Complex formation	T244	T245	model error	FP
23022657_23	Complex formation	T1	T2	model error	FP
23247405_9	Complex formation	T101	T1	model error	FP
23247405_9	Complex formation	T101	T104	model error	FP
25916855_48	Positive regulation	T161	T160	model error	FP
18794358	Catalysis of acetylation	T1	T3	model error	FN
18794358	Catalysis of acetylation	T2	T3	model error	FN
12730668	Catalysis of deacetylation	T5	T4	model error	FN
11341840	Catalysis of methylation	T7	T6	model error	FN

PMID	Relationship type	Entity 1	Entity 2	Error type	FP/FN
11341840	Catalysis of methylation	T17	T16	model error	FN
15145825	Catalysis of methylation	T7	T6	model error	FN
15145825	Catalysis of methylation	T15	T18	model error	FN
15145825	Catalysis of methylation	T14	T13	model error	FN
17018294	Catalysis of SUMOylation	T5	T6	model error	FN
10770935	Complex formation	T3	T7	model error	FN
11099047	Complex formation	T8	T9	model error	FN
18794358	Complex formation	T19	T21	model error	FN
18794358	Complex formation	T19	T22	model error	FN
19637179_9	Complex formation	T14	T29	model error	FN
10763828	Negative regulation	T33	T21	model error	FN
22194618	Negative regulation	T5	T28	model error	FN
22194618	Negative regulation	T5	T7	model error	FN
19637179_9	Negative regulation	T19	T10	model error	FN
10763828	Negative regulation	T16	T17	model error	FN
12548019	Positive regulation	T11	T8	model error	FN
23240008	Regulation of transcription	T3	T4	model error	FN
11099047	Regulation	T20	T21	model error	FN
9857197	Regulation	T1	T26	model error	FN
14752096	Regulation	T1	T7	model error	FN
18094119	Regulation	T26	T22	model error	FN
20858899	Regulation	T32	T34	model error	FN
22958824_7	Regulation	T5	T4	model error	FN
25819761_21	Regulation	T340	T342	model error	FN
18094119	Regulation	T24	T22	model error	FN
20406818	Regulation	T16	T17	model error	FN
25916855_48	Regulation	T161	T160	model error	FN
18094119	Regulation	T25	T22	model error	FN
15031289	Catalysis of phosphorylation	T15	T16	rare keyword	FN
2002555	Complex formation	T5	T4	rare keyword	FN
14981544	Complex formation	T27	T28	rare keyword	FN
18624796	Complex formation	T20	T21	rare keyword	FN
18624796	Complex formation	T19	T21	rare keyword	FN
20129058	Complex formation	T14	T15	rare keyword	FN
22086907_3	Complex formation	T5	T4	rare keyword	FN
22219378_47	Complex formation	T704	T706	rare keyword	FN
26984517_50	Complex formation	T304	T305	rare keyword	FN
26984517_50	Complex formation	T306	T305	rare keyword	FN

PMID	Relationship type	Entity 1	Entity 2	Error type	FP/FN
26984517_50	Complex formation	T307	T309	rare keyword	FN
26984517_50	Complex formation	T311	T312	rare keyword	FN
26984517_50	Complex formation	T311	T313	rare keyword	FN
18094119	Negative regulation	T13	T14	rare keyword	FN
18094119	Negative regulation	T24	T23	rare keyword	FN
18094119	Negative regulation	T25	T23	rare keyword	FN
18094119	Negative regulation	T26	T23	rare keyword	FN
22086907_3	Negative regulation	T9	T14	rare keyword	FN
12548019	Positive regulation	T23	T11	rare keyword	FN
14536086	Positive regulation	T1	T2	rare keyword	FN
14536086	Positive regulation	T27	T29	rare keyword	FN
14536086	Positive regulation	T26	T29	rare keyword	FN
17615152	Positive regulation	T19	T7	rare keyword	FN
17615152	Positive regulation	T22	T7	rare keyword	FN
21236256	Positive regulation	T8	T20	rare keyword	FN
21236256	Positive regulation	T8	T21	rare keyword	FN
27578797	Positive regulation	T6	T7	rare keyword	FN
29666278	Positive regulation	T6	T19	rare keyword	FN
23615448_14	Positive regulation	T245	T246	rare keyword	FN
22086907_3	Regulation of degradation	T9	T14	rare keyword	FN
17615152	Regulation of gene expression	T19	T7	rare keyword	FN
17615152	Regulation of gene expression	T22	T7	rare keyword	FN
17572495	Regulation of transcription	T33	T35	rare keyword	FN
17572495	Regulation of transcription	T34	T35	rare keyword	FN
21554248	Regulation of transcription	T1	T45	rare keyword	FN
21554248	Regulation of transcription	T35	T42	rare keyword	FN
21554248	Regulation of transcription	T8	T42	rare keyword	FN
7524088	Regulation	T14	T10	rare keyword	FN
7524088	Regulation	T14	T11	rare keyword	FN
7524088	Regulation	T14	T12	rare keyword	FN
10446169	Regulation	T5	T6	rare keyword	FN
10446169	Regulation	T4	T6	rare keyword	FN
17018294	Regulation	T3	T1	rare keyword	FN
18094119	Regulation	T19	T18	rare keyword	FN
20129058	Regulation	T3	T4	rare keyword	FN
20129058	Regulation	T17	T12	rare keyword	FN
20129058	Regulation	T20	T2	rare keyword	FN
20368621	Regulation	T3	T4	rare keyword	FN

PMID	Relationship type	Entity 1	Entity 2	Error type	FP/FN
20368621	Regulation	T5	T4	rare keyword	FN
20512148	Regulation	T28	T27	rare keyword	FN
27578797	Regulation	T4	T22	rare keyword	FN
27578797	Regulation	T5	T23	rare keyword	FN
27578797	Regulation	T19	T24	rare keyword	FN
27578797	Regulation	T8	T19	rare keyword	FN
23615448_14	Regulation	T253	T254	rare keyword	FN
23615448_14	Regulation	T261	T262	rare keyword	FN
9008702_22	Regulation	T104	T108	rare keyword	FN

Supplementary Section 8: Error confusion for False Positives in the RegulaTome test set

Confusion category	#FP
Negative predicted as Complex formation	***244***
Negative predicted as Regulation	***156***
Negative predicted as Positive Regulation	***130***
Negative predicted as Negative Regulation	***113***
Negative predicted as Regulation of transcription	***44***
Negative predicted as Catalysis of ubiquitination	***34***
Negative predicted as Regulation of degradation	***33***
Regulation predicted as Positive Regulation	26
Positive regulation predicted as Regulation	19
Negative predicted as Catalysis of phosphorylation	***15***
Regulation of transcription predicted as Regulation of gene expression	13
Negative predicted as Catalysis of methylation	***14***
Negative predicted as Regulation of gene expression	***16***
Negative predicted as Catalysis of dephosphorylation	***12***
Regulation predicted as Negative Regulation	12
Negative predicted as Catalysis of acetylation	***11***
Complex formation predicted as Regulation	10
Regulation of gene expression predicted as Regulation of transcription	10
Negative regulation predicted as Regulation	9
Negative predicted as Catalysis of posttranslational modification	***7***
Negative regulation predicted as Positive Regulation	7
Positive regulation predicted as Negative Regulation	7
Catalysis of posttranslational modification predicted as Complex formation	6
Regulation predicted as Regulation of transcription	6
Catalysis of posttranslational modification predicted as Catalysis of phosphorylation	5
Catalysis of posttranslational modification predicted as Catalysis of ubiquitination	5
Regulation of translation predicted as Regulation of transcription	5
Regulation predicted as Complex formation	5
Catalysis of other small molecule conjugation or removal predicted as Catalysis of demethylation	4
Complex formation predicted as Negative regulation	4
Complex Formation predicted as Positive Regulation	4
Negative predicted as Catalysis of deacetylation	***4***
Negative predicted as Catalysis of palmitoylation	***4***
Negative regulation predicted as Complex formation	4

Confusion category	#FP
Catalysis of phosphorylation predicted as Catalysis of dephosphorylation	3
Catalysis of small protein conjugation predicted as Catalysis of SUMOylation	3
Negative predicted as Catalysis of demethylation	***3***
Regulation of degradation predicted as Negative Regulation	3
Regulation predicted as Catalysis of posttranslational modification	3
Regulation predicted as Regulation of gene expression	1
Regulation predicted as Regulation (reverse)	3
Negative Regulation predicted as Negative Regulation (reverse)	2
Catalysis of acetylation predicted as Positive regulation	2
Negative predicted as Catalysis of ADP-ribosylation	***2***
Negative predicted as Catalysis of glycosylation	***2***
Negative predicted as Catalysis of SUMOylation	***2***
Other catalysis of small molecule conjugation predicted as Catalysis of glycosylation	2
Catalysis of acylation predicted as Catalysis of palmitoylation	1
Catalysis of demethylation predicted as Catalysis of deacetylation	1
Catalysis of demethylation predicted as Positive regulation	1
Catalysis of glycosylation predicted as Negative Regulation	1
Catalysis of methylation predicted as Negative regulation	1
Catalysis of neddylation predicted as Complex formation	1
Catalysis of posttranslational modification predicted as Regulation	1
Catalysis of ubiquitination predicted as Catalysis of small protein conjugation	1
Negative regulation predicted as Regulation of gene expression	1
Other catalysis of small protein removal predicted as Catalysis of deubiquitination	1
Positive regulation predicted as Complex formation	1
Regulation of degradation predicted as Complex formation	1
Regulation of transcription predicted as Catalysis of methylation	1
Regulation of transcription predicted as Positive Regulation	1
Regulation predicted as Catalysis of deacetylation	1
Regulation predicted as Other catalysis of small molecule conjugation	1
Regulation predicted as Regulation of degradation	1
Regulation of translation predicted as Regulation of gene expression	2
**Total**	**1048**

Supplementary Section 9: Error confusion for False Negatives in the RegulaTome test set

Confusion category	#FN
Complex formation predicted as Negative	***237***
Regulation predicted as Negative	***201***
Positive Regulation predicted as Negative	***137***
Negative regulation predicted as Negative	***128***
Regulation of transcription predicted as Negative	***52***
Regulation of gene expression predicted as Negative	***38***
Catalysis of phosphorylation predicted as Negative	***30***
Regulation predicted as Positive Regulation	26
Catalysis of posttranslational modification predicted as Negative	***21***
Positive regulation predicted as Regulation	19
Catalysis of ubiquitination predicted as Negative	***17***
Catalysis of methylation predicted as Negative	***15***
Regulation of transcription predicted as Regulation of gene expression	14
Catalysis of dephosphorylation predicted as Negative	***13***
Regulation predicted as Negative Regulation	12
Catalysis of acetylation predicted as Negative	***10***
Complex formation predicted as Regulation	9
Negative regulation predicted as Regulation	9
Regulation of degradation predicted as Negative	***9***
Regulation of gene expression predicted as Regulation of transcription	10
Catalysis of SUMOylation predicted as Negative	***7***
Negative regulation predicted as Positive regulation	7
Positive regulation predicted as Negative Regulation	7
Regulation predicted as Complex formation	7
Catalysis of deneddylation predicted as Negative	***6***
Catalysis of glycosylation predicted as Negative	***6***
Catalysis of posttranslational modification predicted as Complex formation	6
Regulation predicted as Regulation of transcription	6
Catalysis of palmitoylation predicted as Negative	***5***
Catalysis of posttranslational modification predicted as Catalysis of phosphorylation	5
Catalysis of posttranslational modification predicted as Catalysis of ubiquitination	5
Complex formation predicted as Positive Regulation	5
Negative regulation predicted as Complex Formation	5
Regulation of translation predicted as Regulation of transcription	5
Catalysis of ADP-ribosylation predicted as Negative	***4***
Catalysis of deacetylation predicted as Negative	***4***

Confusion category	#FN
Catalysis of other small molecule conjugation or removal predicted as Catalysis of demethylation	4
Complex formation predicted as Negative regulation	4
Catalysis of deubiquitination predicted as Negative	***3***
Catalysis of phosphorylation predicted as Catalysis of dephosphorylation	3
Catalysis of small protein conjugation predicted as Catalysis of SUMOylation	3
Regulation of degradation predicted as Negative Regulation	3
Regulation predicted as Catalysis of posttranslational modification	3
Regulation predicted as Regulation (reverse)	3
Catalysis of acetylation predicted as Positive regulation	2
Catalysis of other small molecule conjugation or removal predicted as Negative	***2***
Catalysis of small protein conjugation or removal predicted as Negative	***2***
Negative Regulation predicted as Negative Regulation (reverse)	2
Negative regulation predicted as Regulation of gene expression	1
Other catalysis of small molecule conjugation predicted as Catalysis of glycosylation	2
Regulation of gene expression predicted as Complex formation	2
Regulation of translation predicted as Regulation of gene expression	2
Catalysis of acylation predicted as Catalysis of palmitoylation	1
Catalysis of demethylation predicted as Catalysis of deacetylation	1
Catalysis of demethylation predicted as Negative	***1***
Catalysis of glycosylation predicted as Negative Regulation	1
Catalysis of methylation predicted as Negative regulation	1
Catalysis of neddylation predicted as Complex formation	1
Catalysis of phosphoryl group conjugation or removal predicted as Negative	***1***
Catalysis of posttranslational modification predicted as Regulation	1
Catalysis of small protein conjugation predicted as Negative	***1***
Complex formation predicted as Regulation of transcription	1
Negative regulation predicted as Regulation of transcription	1
Other catalysis of small molecule conjugation predicted as Catalysis of posttranslational modification	1
Other catalysis of small protein conjugation predicted as Negative	***1***
Other catalysis of small protein removal predicted as Catalysis of deubiquitination	1
Positive regulation predicted as Catalysis of palmitoylation	1
Positive regulation predicted as Complex formation	1
Regulation of degradation predicted as Complex formation	1
Regulation of transcription predicted as Catalysis of methylation	1
Regulation of transcription predicted as Positive regulation	1
Regulation predicted as Catalysis of deacetylation	1
Regulation predicted as Other catalysis of small molecule conjugation	1
Regulation predicted as Regulation of gene expression	1
**Total**	**1,160**

Supplementary Section 10: Regulatory network evaluation

Correctly assigned directed interactions

$TP_{\text{Catalysis of deubiquitination}}$ + $TP_{\text{Catalysis of demethylation}}$ + $TP_{\text{Catalysis of ubiquitination}}$ + $TP_{\text{Catalysis of phosphorylation}}$ + $TP_{\text{Catalysis of dephosphorylation}}$ + $TP_{\text{Catalysis of methylation}}$ + $TP_{\text{Catalysis of neddylation}}$ + $TP_{\text{Negative regulation}}$ + $TP_{\text{Positive regulation}}$ + $TP_{\text{Regulation of degradation}}$ + $TP_{\text{Regulation of transcription}}$ + $TP_{\text{Catalysis of acetylation}}$ + $TP_{\text{Catalysis of palmitoylation}}$ + $TP_{\text{Regulation of gene expression}}$ + $TP_{\text{Catalysis of SUMOylation}}$ + $TP_{\text{Catalysis of deacetylation}}$ + $TP_{\text{Regulation}}$ + $TP_{\text{Catalysis of ADP-ribosylation}}$ + $TP_{\text{Other catalysis of small molecule conjugation}}$ + $TP_{\text{Catalysis of small protein conjugation}}$ + $TP_{\text{Catalysis of posttranslational modification}}$ + $TP_{\text{Catalysis of glycosylation}}$ + Regulation predicted as Positive Regulation + Positive regulation predicted as Regulation + Regulation of transcription predicted as Regulation of gene expression + Regulation predicted as Negative Regulation + Regulation of gene expression predicted as Regulation of transcription + Negative regulation predicted as Regulation + Negative regulation predicted as Positive Regulation + Positive regulation predicted as Negative Regulation + Regulation predicted as Regulation of transcription + Catalysis of posttranslational modification predicted as Catalysis of phosphorylation + Catalysis of posttranslational modification predicted as Catalysis of ubiquitination + Regulation of translation predicted as Regulation of transcription + Catalysis of other small molecule conjugation or removal predicted as Catalysis of demethylation + Catalysis of phosphorylation predicted as Catalysis of dephosphorylation + Catalysis of small protein conjugation predicted as Catalysis of SUMOylation + Regulation of degradation predicted as Negative Regulation + Regulation predicted as Catalysis of posttranslational modification + Regulation predicted as Regulation of gene expression + Catalysis of acetylation predicted as Positive regulation + Other catalysis of small molecule conjugation predicted as Catalysis of glycosylation + Catalysis of acylation predicted as Catalysis of palmitoylation + Catalysis of demethylation predicted as Catalysis of deacetylation + Catalysis of demethylation predicted as Positive regulation + Catalysis of glycosylation predicted as Negative Regulation + Catalysis of methylation predicted as Negative regulation + Catalysis of posttranslational modification predicted as Regulation + Catalysis of ubiquitination predicted as Catalysis of small protein conjugation + Negative regulation predicted as Regulation of gene expression + Other catalysis of small protein removal predicted as Catalysis of deubiquitination + Regulation of transcription predicted as Catalysis of methylation + Regulation of transcription predicted as Positive Regulation + Regulation predicted as Catalysis of deacetylation + Regulation predicted as Other catalysis of small molecule conjugation + Regulation predicted as Regulation of degradation + Regulation of translation predicted as Regulation of gene expression = 12 + 16 + 77 + 65 + 32 + 35 + 1 + 263 + 276 + 36 + 102 + 17 + 7 + 48 + 7 + 5 + 223 + 2 + 1 + 1 + 6 + 1 + 26 + 19 + 13 + 12 + 10 + 9 + 7 + 7 + 6 + 5 + 5 + 5 + 4 + 3 + 3 + 3 + 3 + 1 + 2 + 2 + 1 + 1 + 1 + 1 + 1 + 1 + 1 + 1 + 1 + 1 + 1 + 1 + 1 + 1 + 2 = 1394

Failed to assign a directed interaction, where there should be one

Regulation predicted as Negative + Positive Regulation predicted as Negative + Negative regulation predicted as Negative + Regulation of transcription predicted as Negative + Regulation of gene expression predicted as Negative + Catalysis of phosphorylation predicted as Negative + Catalysis of posttranslational modification predicted as Negative + Catalysis of ubiquitination predicted as Negative + Catalysis of methylation predicted as Negative + Catalysis of dephosphorylation predicted as Negative + Catalysis of acetylation predicted as Negative + Regulation of degradation predicted as Negative + Catalysis of SUMOylation predicted as Negative + Regulation predicted as Complex formation + Catalysis of deneddylation predicted as Negative + Catalysis of glycosylation predicted as Negative + Catalysis of posttranslational modification predicted as Complex formation + Catalysis of palmitoylation predicted as Negative + Negative regulation predicted as Complex Formation + Catalysis of ADP-ribosylation predicted as Negative + Catalysis of deacetylation predicted as Negative + Catalysis of deubiquitination predicted as Negative + Catalysis of other small molecule conjugation or removal predicted as Negative + Catalysis of small protein conjugation or removal predicted as Negative + Regulation of gene expression predicted as Complex formation + Catalysis of demethylation predicted as Negative + Catalysis of neddylation predicted as Complex formation + Catalysis of phosphoryl group conjugation or removal predicted as Negative + Catalysis of small protein conjugation predicted as Negative + Other catalysis of small protein conjugation predicted as Negative + Positive regulation predicted as Complex formation + Regulation of degradation predicted as Complex formation = 201 + 137 + 128 + 52 + 38 + 30 + 21 + 17 + 15 + 13 + 10 + 9 + 7 + 7 + 6 + 6 + 6 + 5 + 5 + 4 + 4 + 3 + 2 + 2 + 2 + 1 + 1 + 1 + 1 + 1 + 1 + 1 = 737

Assigned a directed interaction, where there should be none

Negative predicted as Regulation + Negative predicted as Positive Regulation + Negative predicted as Negative Regulation + Negative predicted as Regulation of transcription + Negative predicted as Catalysis of ubiquitination + Negative predicted as Regulation of degradation + Negative predicted as Catalysis of phosphorylation + Negative predicted as Catalysis of methylation + Negative predicted as Regulation of gene expression + Negative predicted as Catalysis of dephosphorylation + Negative predicted as Catalysis of acetylation + Complex formation predicted as Regulation + Negative predicted as Catalysis of posttranslational modification + Complex formation predicted as Negative regulation + Complex Formation predicted as Positive Regulation + Negative predicted as Catalysis of deacetylation + Negative predicted as Catalysis of palmitoylation + Negative predicted as Catalysis of demethylation + Negative predicted as Catalysis of ADP-ribosylation + Negative predicted as Catalysis of glycosylation + Negative predicted as Catalysis of SUMOylation = 156 + 130 + 113 + 44 + 34 + 33 + 15 + 14 + 16 + 12 + 11 + 10 + 7 + 4 + 4 + 4 + 4 + 3 + 2 + 2 + 2 = 620

Assigned a directed interaction, but the direction is wrong

Regulation predicted as Regulation (reverse) + Negative Regulation predicted as Negative Regulation (reverse) = 3+2 = 5

Correctly assigned a sign

$TP_{\text{Negative regulation}} + TP_{\text{Positive regulation}}$ = 276 + 263 = 539

Failed to assign a sign (positive or negative), where there should be one

Positive regulation predicted as Regulation + Negative regulation predicted as Regulation + Negative regulation predicted as Regulation of gene expression + Negative regulation predicted as Regulation of transcription + Positive regulation predicted as Catalysis of palmitoylation = 19 + 9 + 1 + 1 + 1 = 31

Assigned a sign, where there should be none

Regulation predicted as Positive Regulation + Regulation predicted as Negative Regulation + Regulation of degradation predicted as Negative Regulation + Catalysis of acetylation predicted as Positive regulation + Catalysis of demethylation predicted as Positive regulation + Catalysis of glycosylation predicted as Negative Regulation + Catalysis of methylation predicted as Negative regulation + Regulation of transcription predicted as Positive Regulation = 26 + 12 + 3 + 2 + 1 + 1 + 1 + 1 = 47

Assigned a sign, but the sign is wrong

Negative Regulation predicted as Positive Regulation + Positive Regulation predicted as Negative Regulation = 7 + 7 = 14
